# Peripheral Odontogenic Keratocyst of the Gingiva: A Systematic Review of the Literature and Case Report

**DOI:** 10.3390/diagnostics15202616

**Published:** 2025-10-16

**Authors:** Marta Forte, Alfonso Manfuso, Giuseppe D’Albis, Giulia Cianciotta, Eliano Cascardi, Grazia Pinto, Giuseppe Ingravallo, Gianfranco Favia, Antonio d’Amati, Luisa Limongelli, Saverio Capodiferro

**Affiliations:** 1 Department of Interdisciplinary Medicine, University of Bari “Aldo Moro”, Piazza Giulio Cesare, 11, 70124 Bari, Italy; alfonso.manfuso@policlinico.ba.it (A.M.); cianciottagiulia20@gmail.com (G.C.); pinto.gr.m@gmail.com (G.P.); gianfranco.favia@uniba.it (G.F.); luisa.limongelli@uniba.it (L.L.); saverio.capodiferro@uniba.it (S.C.); 2 Department of Precision and Regenerative Medicine and Ionian Area (DiMePRe-J), University of Bari “Aldo Moro”, Piazza Giulio Cesare, 11, 70124 Bari, Italy; eliano.cascardi@policlinico.ba.it (E.C.); giuseppe.ingravallo@uniba.it (G.I.); antonio.damati@uniba.it (A.d.)

**Keywords:** peripheral odontogenic keratocyst, odontogenic keratocyst, odontogenic cyst, oral cavity, jaws

## Abstract

**Background/Objectives**: Odontogenic keratocysts are benign cysts originating from remnants of the dental lamina, rarely showing peripheral (gingival) localization. In this study, we compiled data on the peripheral variant by reviewing the literature and presenting a new case to establish criteria for accurate differential diagnosis and treatment. **Methods**: A systematic literature review was conducted following the PRISMA flowchart, leading to the collection of existing data on peripheral odontogenic keratocyst. In addition, we present a new case of a 68-year-old female patient referred to our attention for an asymptomatic swelling of the mandible in the premolar area. Radiographic examination revealed a round radiolucency with well-defined borders located between teeth #4.3 and #4.4, surgically removed and diagnosed as a peripheral (gingival) keratocyst. **Results**: Including the herein described new case, 37 cases were reviewed from data literature showing occurrence in the mandible (43.2%) and maxilla (46%)—with 10.8% of cases not stated-, with an age range of 14–83 year old, recurrence rate of 12.5–13.6% (total recurrences/total cases) and median follow-up time of 19 months. **Conclusions**: Data from literature highlights the rarity of odontogenic keratocyst with peripheral (gingival) localization, which can be misleading for differential diagnosis, emphasizing the necessity of histopathological examination as the definitive diagnostic tool for all the cystic lesions of the jaws. The absence of pathognomonic clinical and radiological features, combined with the potential for extraosseous manifestation of odontogenic lesions with high recurrence rates, underscores the importance of complete excision to ensure proper healing and prevent recurrence of odontogenic keratocyst.

## 1. Introduction

Among odontogenic lesions, the odontogenic keratocyst (OKC) is a common occurrence in the oral cavity, typically characterized by its aggressive and locally invasive nature. It originates from the remnants of the dental lamina and constitutes approximately 10–20% of all jaw cysts [1]. Compared to other odontogenic cysts, OKC has a high recurrence rate (up to 62.5%) and exhibits significant local aggressiveness. Approximately 80% of cases occur in the mandibular region, with a notable preference for the posterior area and the mandibular ramus [2]. Due to its asymptomatic nature, OKC is often diagnosed incidentally through routine radiographic examinations, appearing as either a unilocular or multilocular well-defined radiolucency. Particularly in young patients, it may be associated with an impacted tooth in approximately 30% of cases. Histopathological examination reveals that the cystic lumen contains a creamy, straw-colored fluid, mainly composed of keratin granules. Complete surgical excision remains a challenge due to the exceptionally thin epithelial wall.

Although traditionally considered a subtype of OKC, in 2022, the 5th Edition of the World Health Organization Classification of Head and Neck Tumors reclassified the peripheral variant as a distinct entity due to its unique clinical, histopathological, and biological characteristics.

The extraosseous variant, also referred to as the peripheral odontogenic keratocyst (POKC), is exceedingly rare, with only 36 cases described in the literature [3]. Clinically, it typically presents as a fluctuant soft tissue swelling, ranging from 3 mm to 5 cm in size. It may also be associated with infection, sometimes exhibiting purulent exudate. Radiographic imaging is essential for evaluating lesion localization and potential bony involvement. Orthopantomography is sufficient for assessing bony changes; however, a CT scan is required for a more precise delineation of the lesion’s size, borders, and anatomical relationships [2,3,4,5]. While POKC is generally not apparent on radiographs, it may occasionally present as a unilocular or multilocular radiolucent lesion, resembling other odontogenic cysts and neoplasms [3,4,5].

The presence of extraosseous lesions in the oral cavity presents a significant diagnostic challenge for pathologists and surgeons, often necessitating surgical removal. Differential diagnoses should include inflammatory lesions (e.g., inflammatory cysts, pyogenic granulomas, epulis fissuratum, or dental abscesses) and non-inflammatory lesions (e.g., peripheral giant cell granuloma, lateral periodontal cyst [LPC], gingival cyst of the adult [GCA], peripheral ameloblastoma, peripheral ossifying fibroma, mucocele, fibroma, or vascular lesion). Although benign, POKC exhibits a tendency for recurrence, highlighting the importance of accurate diagnosis and appropriate management. This study aims to review the epidemiology, clinical features, diagnosis, histopathological characteristics, treatment options, recurrence rates, and long-term outcomes of POKC. Additionally, a new case with a gingival manifestation is described, contributing further data to guide the differential diagnosis of peripheral lesions in the oral cavity.

## 2. Materials and Methods

### 2.1. Literature Analysis

#### 2.1.1. Protocol and Registration

The study protocol was registered with the International Prospective Register of Systematic Reviews (PROSPERO)—Registration number: 1043819.

#### 2.1.2. Search Strategy

A systematic electronic search was performed based on the PICO criteria (Table 1). The research question was formulated as follows: In adults, what are the clinical and histopathological features (I), in relation to other similar peripheral lesions (C), of peripheral keratocysts (P) that enable a better understanding of recurrence rates and long-term outcomes (O)?

A comprehensive systematic review was conducted by four independent reviewers (M.F., A.M., G.D., and S.C.), covering the period from November 2024 to February 2025. The search was performed using a combination of keywords and Medical Subject Headings (MeSH) terms, including: “Peripheral Odontogenic Keratocyst” OR “POKC” AND “Oral Cavity” OR “Odontogenic Keratocyst” AND “Peripheral.”

**Figure 1:**
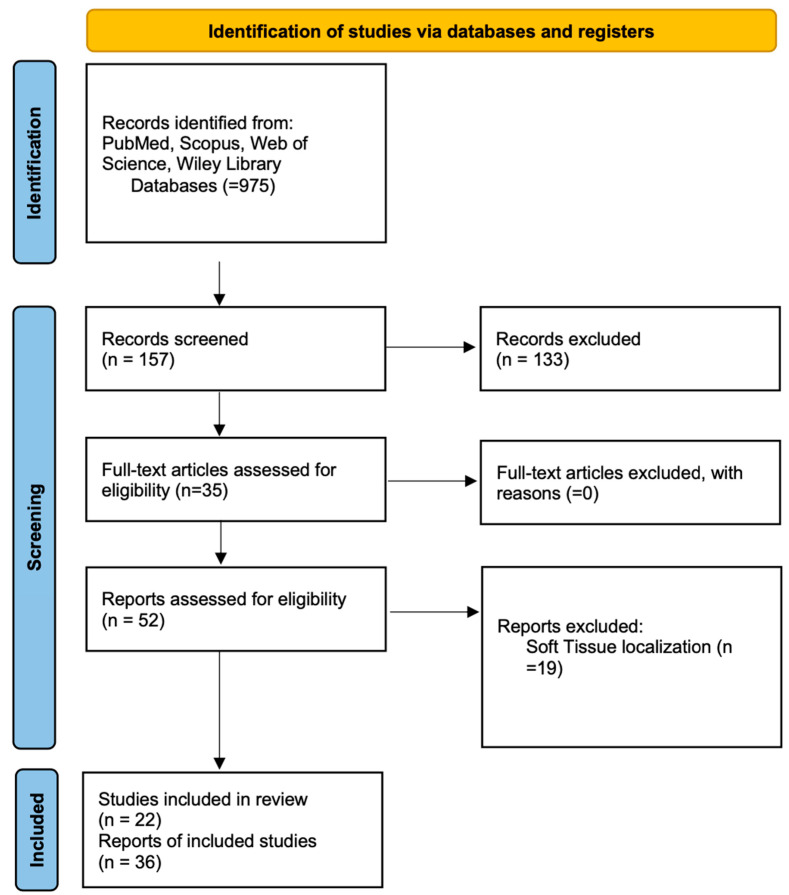
Search results were conducted and reported in accordance with the PRISMA 2020 statement.

Articles published between 1974 and 2024 were evaluated for relevance based on their focus on the clinical, histopathological, or treatment-related aspects of POKC. These included studies reporting cases with a histological diagnosis of POKC, case series, case reports, and reviews. Studies that did not specifically address POKC, those lacking a histological diagnosis of POKC, those based on animal models, or case reports with insufficient data were excluded. This study followed the PRISMA Statement guidelines of Preferred Reporting Items of Systematic Review, as also summarized in Figure 1.

Additionally, cases involving buccal mucosa lesions with immunopathological characteristics consistent with POKC were identified but excluded from the review due to the difficulty in demonstrating their odontogenic nature, according to the opinion of some authors [7,8,9]. In support of this latter theory, we gathered a series of buccal mucosa POKC (Table 2) that were excluded from our review.

## 3. Results

### 3.1. Case Presentation

**Figure 2:**
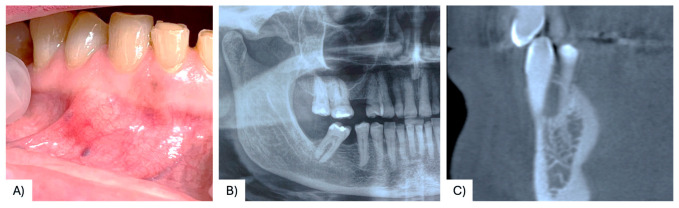
Small swelling of the vestibular gingiva in the region of #43 and #44 (**A**); radiograph showing a roundish radiolucency with radiolucent borders between elements #4.3 and #4.4 (**B**,**C**).

**Figure 3:**
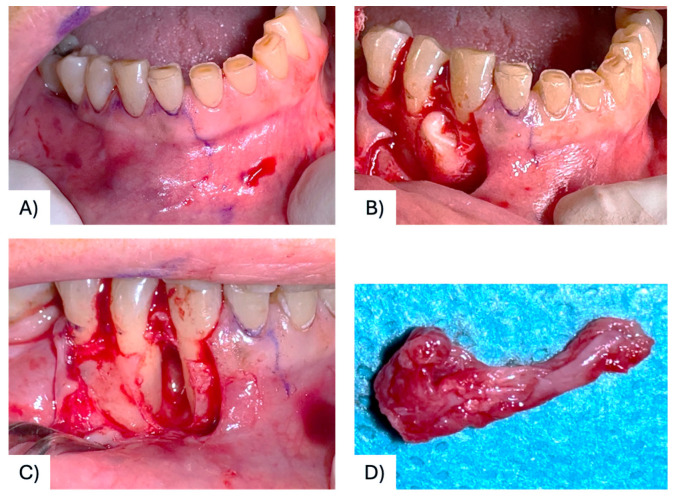
Surgical design of the flap to access the lesion (**A**); mucoperiosteal flap elevation showing the cystic lesion located between #43 and #44 (**B**); intraoperative view after cyst removal and curettage (**C**); The surgical specimen (**D**).

**Figure 4:**
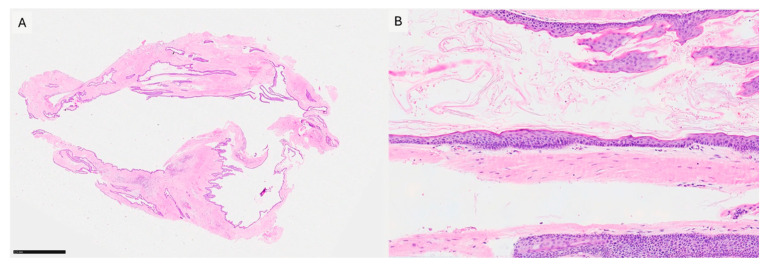
(**A**) Low power magnification showing the overall cystic architecture of the lesion (H&E, 20×). (**B**) High power magnification showing a para-keratinized stratified squamous epithelium with a palisaded basal cell layer surrounding connective tissue (HE, 100×).

**Figure 5:**
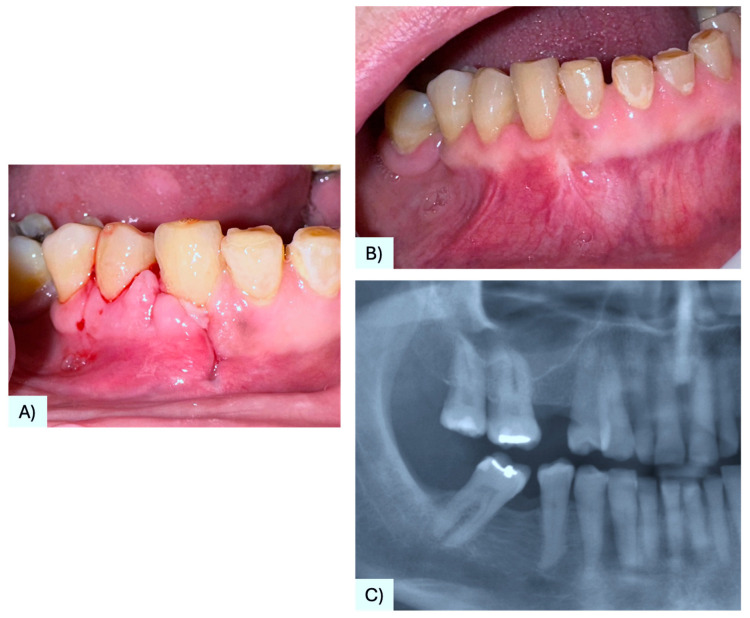
Partial healing of the treated area at 3 weeks clinical follow-up (**A**); complete clinical healing of the soft tissue at 2 months follow-up (**B**); 3 months radiographic follow-up showing no bone alteration, also with healing of the treated site (**C**).

**Figure 6:**
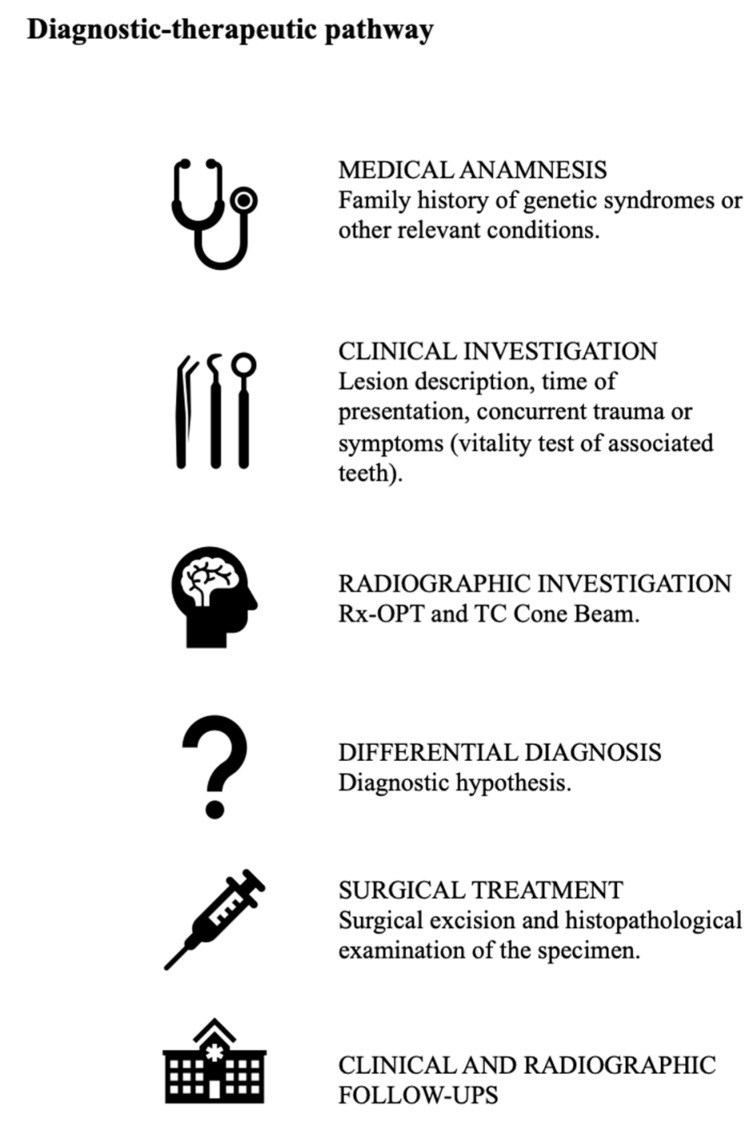
Summary of the current diagnostic-therapeutic pathway.

A 68-year-old female patient presenting with a small, asymptomatic swelling of the gingiva between the right mandibular canine and first premolar was referred to the University of Bari “Aldo Moro.” The patient denied any history of trauma and reported the lesion’s presence for approximately one year. There was no family history of genetic syndromes or other relevant conditions. Intraoral examination revealed a well-defined, firm, asymptomatic lesion measuring approximately 1.5–2 cm in diameter, confined to the vestibular gingival surface in the region of teeth #4.3 and #4.4 (Figure 2A). Both involved teeth responded positively to vitality testing. Radiographic examination showed a round radiopaque lesion with well-defined radiolucent borders between the roots of #4.3 and #4.4 (Figure 2B,C). Under local anesthesia, a mucoperiosteal marginal flap was elevated, showing the entire lesion. During enucleation, the cystic wall ruptured, releasing a dense, straw-colored fluid; following complete lesion removal, significant alveolar bone resorption was noted, with the lesion in contact with the roots (Figure 3A–C). The cavity was meticulously curetted and the involved roots as well, and the flap was sutured with Vicryl 5/0. The specimen was fixed in 10% formalin and sent for histopathological analysis (Figure 3D). Microscopic examination revealed a stratified squamous epithelium with a parakeratotic epithelial surface and a hyperchromatic, palisaded basal cell layer, leading to the diagnosis of POKC (Figure 4). The postoperative course was uneventful, with no complications observed. Clinical and radiological follow-up at three-month intervals showed no recurrence (Figure 5). Figure 6 summarizes the diagnostic and therapeutic pathway.

### 3.2. Literature Analysis

From data research, only 22 articles matched the inclusion criteria; 36 cases were described and summarized in Table 2.

Based on the analyzed data, the literature reports only 37 cases with a histological diagnosis of Peripheral Odontogenic Keratocyst (POKC) from 1975 to 2024. These cases include 19 females, 11 males, and 5 individuals with unspecified gender, with ages ranging from 14 to 83 years old. Maxilla was the most prevalent site of manifestation, with 18 cases, mandible 16 cases, although 4 studies did not specify the site of occurrence. In most cases, no radiographic findings were reported, with only 9 cases showing radio-lucent areas with well-defined margins. Nearly all authors opted for surgical management of the lesion, primarily employing conservative enucleation or excision techniques. During the literature review, existing cases involving lesions of the buccal mucosa with immunopathological characteristics consistent with the diagnosis of POKC were also identified. However, according to several authors [7,8,9], these characteristics alone are not sufficient to classify such lesions as part of the same pathological entity described in this study, due to the ongoing difficulty in demonstrating their odontogenic origin. Supporting this position, a compilation of buccal mucosa POKC cases (summarized in Table 3) was excluded from the final review.

### 3.3. Quality Assessment and Risk of Bias

Quality assessment and risk of bias evaluation were evaluated by two reviewers (M.F. and G.D.) using the Joanna Briggs Institute (JBI) Critical Appraisal Checklist for Case Reports, which included the reported studies (Table 4). In cases of disagreement, a third reviewer (L.L.) was consulted to resolve discrepancies, and discussions continued until a consensus was achieved. This evaluation was assessed through eight questions (Q1–Q8). Q1: Does the case report clearly describe the patient’s age, sex, race, medical history, diagnosis, prognosis, previous treatments, past and current diagnostic test results, and medications? The setting and context; Q2: A good case report will clearly describe the history of the patient, their medical, family and psychosocial history including relevant genetic information, as well as relevant past interventions and their outcomes (CARE Checklist 2013); Q3: The current clinical condition of the patient should be described in detail including the uniqueness of the condition/disease, symptoms, frequency and severity. The case report should also be able to present whether differential diagnoses was considered may also be described; Q4: A reader of the case report should be provided sufficient information to understand how the patient was assessed. It is important that all appropriate tests are ordered to confirm a diagnosis, and therefore, the case report should provide a clear description of various diagnostic tests used (whether a gold standard or alternative diagnostic tests). Photographs or illustrations of diagnostic procedures, radiographs, or treatment procedures are usually presented when appropriate to convey a clear message to readers; Q5: It is important to clearly describe treatment or intervention procedures, as other clinicians will be reading the paper and therefore may enable a clear understanding of the treatment protocol. The report should describe the treatment/intervention protocol in detail; for e.g., in pharmacological management of dental anxiety—the type of drug, route of administration, drug dosage and frequency, and any side effects; Q6: A good case report should clearly describe the clinical condition post-intervention in terms of the presence or lack thereof symptoms. The outcomes of management/treatment when presented as images or figures would help in conveying the information to the reader/clinician. Q7: With any treatment/intervention/drug, there are bound to be some adverse events and, in some cases, they may be severe. It is important that adverse events are clearly documented and described, particularly when a new or unique condition is being treated or when a new drug or treatment is used. In addition, unanticipated events, if any, that may yield new or useful information should be identified and clearly described; Q8: Case reports should summarize key lessons learned from a case in terms of the background of the condition/disease and clinical practice guidance for clinicians when presented with similar cases. Ref [37] Most of the included studies exhibited a low overall risk of bias, indicating solid methodological rigor and reliability of outcomes. However, certain domains—particularly those related to incomplete demographic data, radiographic evaluation and outcomes—revealed moderate concerns, underscoring the need for enhanced transparency and data management in future studies.

## 4. Discussion

OKC is one of the most frequently diagnosed cystic lesions. In 2005, the WHO classified OKC as a benign neoplasm due to the presence of mutations in the Protein Patched Homolog 1 (PTCH) tumor suppressor gene. These mutations have since been identified in non-neoplastic lesions, leading the WHO to reclassify OKC as an odontogenic cyst in 2017. Despite this latest classification, its locally aggressive behavior and invasiveness often require a more radical treatment approach to ensure complete removal and healing without recurrence [5]. OKC with peripheral manifestations represents an extraosseous variant known as POKC, which remains extremely rare and poorly documented in the literature [3].

### 4.1. Pathogenesis

The pathogenesis of POKC remains incompletely understood. It is believed to originate from remnants of the dental lamina or the rests of Malassez within the gingival tissue. These remnants may proliferate, forming a cystic lesion with keratinizing epithelial linings. Unlike central OKCs, POKCs do not invade bone but remain confined to the peripheral gingival tissues. Some studies suggest that genetic mutations may contribute to its development, while other hypotheses propose that local inflammatory stimuli or trauma could act as triggering factors [7,8,9,10,11,12,13,14,15,16,17,18,20,21,22,23,24,25,26,27].

### 4.2. Clinical and Radiographical Features

POKC typically presents as an asymptomatic nodular swelling of soft tissue, with variations in color from white to yellow and consistency ranging from fluctuant to firm. The most commonly reported anatomical site is the vestibular gingiva, with an average size of 0.8 cm (range: 0.15–2.5 cm) [1,2,3,4,5]. Palatal or lingual gingival involvement is rarely documented [23,24]. The lesion may initially appear small but can gradually enlarge over time. Some patients report mild discomfort or tenderness, particularly if secondary infection occurs. In advanced cases, POKC may cause displacement of adjacent teeth, though significant bone destruction is uncommon. POKC is usually not associated with other odontogenic or systemic disorders, although a few cases have been linked to nevoid basal cell carcinoma syndrome (Gorlin-Goltz syndrome). Clinical differential diagnosis should include a variety of odontogenic cysts, neoplasms, and non-odontogenic lesions with similar clinical presentations [8,17]. The main characteristics of POKC differential diagnoses are summarized in Table 4.

### 4.3. Histopathological Features

Distinct histological characteristics enable pathologists to identify POKC. The cystic wall is composed of stratified parakeratinized squamous epithelium with a palisaded basal cell layer. Multiple invaginations of the epithelium may also be observed. The cystic lumen contains a thick, straw-colored, creamy fluid composed of keratin [5,32]. Although histologically similar to its central intraosseous counterpart, some studies have shown that POKC exhibits a lower percentage of microcysts within the fibrous capsule (12.5%) [1]. To date, no standardized immunohistochemical profile for POKC has been established in the literature. However, Lafuente-Ibanez de Mendoza et al., in their description of three cases, found that all exhibited intense CK14 expression and irregular epithelial CK19 expression [1].

### 4.4. Differential Diagnosis of Peripheral Odontogenic Lesion

Given the rarity of POKC, only a limited number of cases have been described in the literature. Its diagnosis requires a combination of clinical, radiological, and histopathological evaluations. Clinical diagnosis is based on the lesion’s gingival location and characteristic presentation as a painless, slow-growing mass. In some cases, radiographic imaging may reveal a small radiolucent area at the lesion site, although bone involvement is typically absent or minimal. Panoramic radiographs may show a soft-tissue mass without bony destruction, distinguishing POKC from the central form of OKC [1,2,3,8,9,19,22,23,24,25,26,27,30,38,39]. Histological examination remains the gold standard for differential diagnosis, as it confirms a cystic structure lined by stratified squamous epithelium with orthokeratinization or parakeratinization. The epithelium typically exhibits a characteristic corrugated appearance and may contain areas of keratin production. The cyst wall is generally devoid of significant inflammation or fibrosis, distinguishing it from other odontogenic lesions [5,32]. The diagnosis of POKC requires careful differentiation from several other lesions that may present with similar clinical and radiological features. These include peripheral giant cell granuloma, gingival cyst of the adult (GCA), adenomatoid odontogenic tumor (AOT), inflammatory odontogenic cyst, lateral periodontal cyst (LPA), and peripheral ameloblastoma [32,33,34,35,36,40,41,42]. The key distinguishing characteristics of these lesions are summarized in Table 5 and Table 6.

In the present case, the lesion’s location between mandibular teeth #4.3 and #4.4, along with a positive vitality test, initially suggested a gingival cyst of the adult (GCA) [40,43] or lateral periodontal cyst (LPA) [44]. However, certain clinical features, particularly the fragility of the cystic wall and the release of a straw-colored fluid upon rupture, were indicative of OKC and have been similarly reported in previous studies [1,3,17,20,26]. Overall, differentiating POKC from other gingival lesions requires a comprehensive clinical, radiological, and histopathological assessment. The key distinguishing features include the presence of keratinized epithelium, lack of bone involvement, and specific histopathological patterns seen in peripheral giant cell granuloma, lateral periodontal cyst, and ameloblastoma. Accurate diagnosis is crucial to determining the most appropriate treatment and ensuring a favorable prognosis. Furthermore, distinguishing intraosseous OKCs from their peripheral counterparts is essential and is based on specific clinical, radiological, and histopathological characteristics, as summarized in Table 6.

### 4.5. Recurrences and Prognosis

According to existing data, POKC has a significantly lower recurrence rate than the intraosseous variant, with recurrence rates reported at 12.5–13.6% (Table 5) [4,28,29,31,45]. The reason for this lower recurrence rate remains unclear, but two hypotheses have been proposed: (1) the greater ease of complete surgical excision at the soft tissue level, and (2) a potentially distinct biological behavior of the peripheral variant compared to its central counterpart, suggesting it may represent a separate pathological entity [4]. Factors contributing to recurrence include incomplete excision, inadequate follow-up, and lesion size at the time of initial treatment. Most recurrences occur within the first five years post-treatment, although long-term follow-up is recommended [19,22,23,25,38,39]. In this study, the patient underwent clinical follow-ups every three months, with no signs of recurrence observed. The prognosis for POKC is generally favorable when complete excision is achieved, with most patients experiencing no further complications. Recurrence, when it occurs, is typically local and does not result in systemic involvement or malignancy [8,9,19,23,24,25,26,27,38,39].

### 4.6. Treatment Modalities

The management of peripheral odontogenic lesions is challenging due to their lack of radiographic appearance and difficulties in differential diagnosis. Surgical excision remains the primary treatment for POKC. The goal is to achieve complete removal with clear margins to prevent recurrence. The surgical approach is often conservative, involving soft tissue excision with minimal bone involvement. In some cases, curettage may be performed, but this carries a higher recurrence risk. Complete removal of the epithelial lining is essential, as incomplete excision is a common cause of recurrence. For larger lesions, adjunctive treatments such as cryotherapy or peripheral ostectomy may be considered, although their use remains controversial. In this case, excisional biopsy combined with thorough curettage ensured a low recurrence rate and complete healing of the surgical site [3,4,8,9,19,23,24,25,26,27,38].

## 5. Conclusions

POKC is a rare, benign odontogenic lesion that presents in the gingival tissue. It typically manifests as a painless, slow-growing mass and requires a combination of clinical, radiological, and histopathological assessments for diagnosis. Surgical excision with clear margins remains the treatment of choice. Although prognosis is generally favorable, long-term follow-up is essential to monitor for recurrence. Future research into molecular and genetic markers may provide further insights into the pathogenesis of POKC and contribute to the development of targeted therapeutic strategies. Nonetheless, the present study is inherently limited by the scarcity of reported case series in the literature, which constrains the ability to draw statistically robust conclusions and limits the generalizability of the findings. This underscores the need for larger, multicenter investigations and systematic data collection to improve diagnostic accuracy, refine treatment protocols, and better understand the biological behavior of this uncommon entity.

## Figures and Tables

**Table 1:** Search strategy according to the PICO criteria [6].

Focused Question (PICO)	What Are the Clinical and Histopathological Features, in Relation to Other Similar Peripheral Lesions, of Peripheral Keratocysts that Will Allow a Better Understanding of Recurrence Rates and Long-Term Outcomes?
Search Strategy Population	Patients affected by peripheral odontogenic keratocyst, with extraosseous localization.
Intervention	Advanced diagnostic approaches and optimal treatment strategies.
Comparison	Differential diagnosis with other peripheral lesions.
Outcome	Clinical and histopathological features, recurrence rates, treatment options, and long-term outcomes
Database Electronic search	PubMed, Web of Science, Scopus and Wiley Library
Selection criteria Inclusion criteria	Studies at all levels of evidence, except expert opinion; Articles published in English reporting cases with histological diagnosis of POKC
Exclusion criteria	Animal studies; in Vitro study; not available articles

**Table 2:** Summary of POKC cases reported in the literature.

*Nr. CASE*	*Author*	*Age*/ *Gender*	*Site*	*Sign*/ *Syntoms*	*Size*	*Radiographic Appearance*	*Surgical Appearance*	*Treatment*	*Outcomes*	*YEAR*
*1*	Stoelinga PJ et al. [10]	NS	Posterior maxilla	NS	NS	NS	NS	NS	NS	1975
*2*	Amos Buchner et al. [11]	NS	NS	Swelling	NS	NS	NS	NS	NS	1979
*3*	Dan Dayan et al. [12]	42 M	Maxilla	Swelling	10 mm	None	Small fenestration	Enucleation, curettage	No evidence after 10 months	1988
*4*	Antoine Chehade et al. [13]	37 M	Anterior Mandible	Swelling, Fluctuant	3 × 3 mm	None	None	NS	NS	1994
*5*	Antoine Chehade et al. [13]	66 F	Posterior Maxilla	Swelling	NS	None	None	NS	NS	1994
*6*	Antoine Chehade et al. [13]	35 F	Posterior Mandible	Mobile nodule	10 mm	None	None	NS	NS	1994
*7*	Antoine Chehade et al. [13]	70 M	Posterior Mandible	Nodule	NS	None	None	NS	Recurred after 7 years	1994
*8*	Antoine Chehade et al. [13]	57 F	Anterior Maxilla	Swelling	7 × 5 mm	None	None	NS	NS	1994
*9*	Antoine Chehade et al. [13]	42 M	Posterior Mandible	NS	NS	None	Focal fenestration	NS	NS	1994
*10*	Fardal and Johannessen [14]	41 F	Mandibular and maxillary gingiva	NS	NS	NS	NS	NS	NS	1994
*11*	Yih, W. et al. [7]	NS	NS	NS	NS	NS	NS	NS	NS	2000
*12*	Yih, W. et al. [7]	NS	NS	NS	NS	NS	NS	NS	NS	2000
*13*	Yih, W. et al. [7]	NS	NS	NS	NS	NS	NS	NS	NS	2000
*14*	F. Ide et al. [15]	38 F	Mandible gingiva of #3.2	Asymptomatic fluctuant nodule	3 mm	None	None	Excision	No evidence after 5 years	2002
*15*	F. Ide et al. [15]	46 F	Mandible, gingiva of #4.4	Asymptomatic fluctuant nodule	5 mm	None	None	Excision	No evidence after 6 years	2002
*16*	A.C. Chi et al. [16]	81 F	Maxilla, gingiva between #2.1, #2.2	Asymptomatic fluctuant nodule	10 mm	None	Superficial resorption	Enucleation, curettage	Recurrence after 6 months, re-excised, No evidence after 3 months	2005
*17*	A.C. Chi et al. [16]	64 F	Mandible, gingiva between #3.4, #3.5	Asymptomatic nodule	15 mm	None	NS	Enucleation	No evidence after 21 months	2005
*18*	R.D. Preston et al. [17]	83 F	Maxilla, gingiva between #2.3, #2.4	Nodule	7 mm	None	Superficial resorption	Enucleation, curettage	No evidence after 6 months	2005
*19*	E. Mozaffari et al. [18]	82 F	Mandible, gingiva of #3.5	Swelling with persistent mild pain	7 × 5 mm	Radiolucency with adjacent radiopaque shadow	NS	Excisional biopsy	NS	2007
*20*	Usuii H. et al. [19]	53 M	Maxilla, gingiva of #2.3	Asymptomatic fluctuant swelling	6 mm	NS	Alveolar bone resorption	Excision	No evidence after 6 years	2007
*21*	S E S Faustino et al. [20]	57 F	Mandible, gingiva between #3.4, #3.5	Asymptomatic non-mobile nodule	5 mm	Radiolucency with a defined margin area	Cortical resorption	Enucleation, curettage	Recurrence after 1 year, re-enucleated No evidence to date	2008
*22*	F. Ide et al. [21]	53 M	Mandible, gingiva between #3.2, #3.3	Fluctuant nodule	6 mm	Radiolucency	Superficial resorption	NS	No evidence after 7 years	2008
*23*	Jinbu, Y et al. [22]	63 M	Mandible, gingiva of #4.5	Painful, fluctuant nodule	15 mm	Radiolucency with ill-defined margin area	Alveolar bone resorption	Resection	No evidence after 1 year	2009
*24*	H. Vij et al. [23]	56 M	Maxilla, Hard palate gingiva from #2.1 to #4.4	Discharging swelling	25 × 20 mm	None	None	Excisional biopsy	NS	2011
*25*	K. Sakamoto et al. [24]	24 F	Mandible, gingiva of #4.3	Pigmented asymptomatic papule	3 mm	None	None	Excisional biopsy	NS	2014
*26*	María del Carmen Vázquez-Romero et al. [2]	32 M	Maxilla, gingiva between #2.2, #2.3	Asymptomatic fluctuant nodule	4 mm	Hypodense lesion (CBCT)	Cortical resorption	Excisional biopsy, Curettage, Bone drill	No evidence after 1 year	2017
*27*	B.T. Rodrigues et al. [8]	43 F	Maxilla, gingiva between #1.5, #1.6	Asymptomatic nodule	15 mm	None	Superficial resorption	Excisional biopsy	No evidence after 4 years	2020
*28*	B.T. Rodrigues et al. [8]	63 F	Mandible, gingiva between #3.1, #4.1	Asymptomatic elevated lesion	10 mm	None	None	Excisional biopsy	Not returned for follow-ups	2020
*29*	Lafuente-Ibáñez de Mendoza et al. [1]	61 F	Maxilla, gingiva between #2.1, #2.2	Asymptomatic fluctuant nodule	10 mm	None	NS	Excision	No evidence after 20 months	2022
*30*	Lafuente-Ibáñez de Mendoza et al. [1]	74 F	Mandible, gingiva between #3.3, #3.4	Fluctuant nodule	15 mm	None	NS	Excision	No evidence after 4 years	2022
*31*	Lafuente-Ibáñez de Mendoza et al. [1]	14 M	Maxilla, gingiva between #2.4, #2.5	Asymptomatic nodule	20 mm	None	NS	Excision	Already recurred after 2 years, No evidence after 12 months	2022
*32*	Lado Lako Loro et al. [25]	71 M	Maxilla, gingiva between #1.3, #1.4	Asymptomatic nodule	10 mm	Radiolucency with a defined margin area	Alveolar bone resorption	Excision, curettage	No evidence after 10 months	2023
*33*	Lado Lako Loro et al. [25]	33 F	Maxilla, gingiva between #1.3, #1.5	Asymptomatic nodule	3 mm	None	Alveolar bone resorption	Excision	No evidence after 10 months	2023
*34*	Brooks JK et al. [3]	70 F	Maxilla, gingiva between #1.2, #1.3	Non-mobile papule	4 mm	Radiolucency with a defined margin area	Bone defect	Enucleation	Recurred after 8 months, re-enucleated, recurred after 6 months, Re-enucleated, no further evidence after 4 months	2024
*35*	Kinaia et al. [19]	60 F	Mandible; gingi val tissue between #3.1–3.2	Asymptomatic swelling	10 × 9 × 5 mm	Radiolucency with a defined margin area	Loss of the cortical plate	Excisional biopsy of the cystic lesion combined with guided tissue regeneration	No recurrences at 6 months follow-up	2024
*36*	Kinaia et al. [19]	62 M	Maxilla; gingiva betwee#14–15	Asymptomatic swelling	5 × 8 mm	Diffuse radiolucency	No bone alteration	Excisional biopsy of the cystic lesion combined with guided tissue regeneration	No recurrences at 36 months follow-up	2024
*37*	**Present case**	68 F	Maxilla, between #4.3, #4.4	Asymptomatic nodule	15 mm	Radiolucency with a defined margin area	Alveolar bone resorption	Excisional biopsy, curettage	No recurrence at 6 months follow-up	2025

NS: not stated.

**Table 3:** OKC cases localized in soft tissues and excluded from our study.

*CASE*	*AUTHOR*	*AGE*/*GENDER*	*SITE*	*SIGN*/*SYMPTOMS*	*SIZE*	*Radiographic Appearance*	*Surgical Appearance*	*Treatment*	*Outcome*	*YEAR*
*1*	Precheur and Krolls [9]	59 M	Left cheek	Firm, slightly tender, mobile, 3–4 cm mass	NS	NS	NS	Incisional biopsy	NS	2009
*2*	Precheur and Krolls [9]	60 M	Left cheek	Painless Swelling	3 × 2 × 2 cm	NS	NS	Excision	No recurrences	2010
*3*	Gröbe A et al. [26]	52 M	Right buccal mucosa	Asymptomatic fluctuant nodule	20 × 20 mm	None	None	Excision	No evidence after 4 months	2012
*4*	Yamamoto K et al. [27]	74 M	Right buccal mucosa	Asymptomatic fluctuant nodule	30 × 25 mm	Defined margin lesion (CBCT)	None	Excision	No evidence after 4 years	2013
*5*	Kaminagakura E et al. [28]	NS	Left buccal mucosa	NS	NS	NS	NS	NS	No recurrences after 12 years	2013
*6*	Abé T. et al. [29]	46 M	Left temporalis muscle	Painful fluctuant nodule	21 mm	Loculated lesion (CBCT)	Bone resorption	Excision	No evidence after 12 years	2014
*7*	Zhu L et al. [25]	44 M	Soft palate	Asymptomatic nodule	30 × 40 mm	NS	NS	Resection	NS	2014
*8*	Zhu L et al. [25]	69 M	Right buccal mucosa	Asymptomatic non-mobile nodule	20 mm	NS	NS	Resection	NS	2014
*9*	Makarla et al. [30]		Right buccal space	NS	NS	NS	NS	NS	No after 24 months	2015
*10*	Witteveen et al. [31]	NS	Right buccal space	NS	NS	NS	NS	NS	No recurrence after 4 years	2018
*11*	Witteveen et al. [31]	NS	Left buccal space	NS	NS	NS	NS	NS	No recurrence after 1 year	2018
*12*	Beena et al. [32]	NS	Right buccal space	NS	NS	NS	NS	NS	No recurrences after 6 months	2020
*13*	Shatur A et al. [5]	76 M	Retromolar trigone	Painful fluctuant swelling	50 × 40 mm	None	None	Excisional biopsy	NS	2021
*14*	Watanabe et al. [33]	NS	Right buccal space	NS	NS	NS	NS	NS	Two recurrences after 4 years	2022
*15*	Mustakim et al. [34]	NS	Massetere muscle	NS	NS	NS	NS	NS	No recurrences after 5 years	2022
*16*	María Hornillos-de Villota et al. [4]	58 F	Soft tissue of the posterior margin of the mandibular ramus	NS	26 × 19 mm	Loculated lesion (CBCT)	NS	Cystectomy by cervical approach	Already occurred twice, no evidence after 3 months	2023
*17*	Kochaji et al. [35]	17 M	Right cheek	Painless swelling	27 × 15 × 10 mm	None	NS	Biopsy	6 months	2023
*18*	Thayanne Oliveira de Freitas Gonçalves et al. [36]	58 M	Right buccal mucosa	Painful swelling	5 cm	None	NS	Biopsy	No recurrences at 18 months	2025
*19*	Thayanne Oliveira de Freitas Gonçalves et al. [36]	44 M	Left buccal mucosa	Painful swelling	NS	None	NS	Biopsy	No recurrences at 12 months	2025
*20*	Thayanne Oliveira de Freitas Gonçalves et al. [36]	74 F	Left buccal mucosa	Painful swelling	13 mm	None	NS	Biopsy	Lost follow-up	2025

**Table 4:** Risk of Bias assessment. [Joanna Briggs Institute (JBI) Critical Appraisal Checklist for Case Reports].

	Q1	Q2	Q3	Q4	Q5	Q6	Q7	Q8
Stoelinga PJ et al. [10]	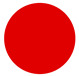	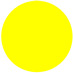	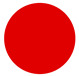	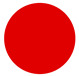	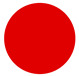	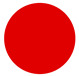	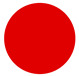	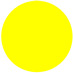
Amos Buchner et al. [11]	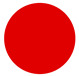	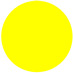	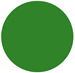	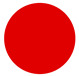	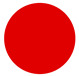	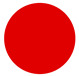	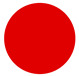	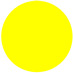
Dan Dayan et al. [12]	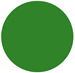	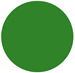	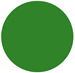	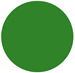	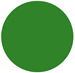	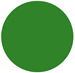	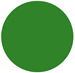	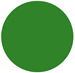
Antoine Chehade et al. [13]	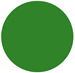	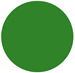	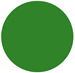	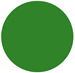	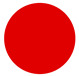	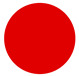	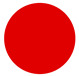	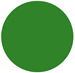
Antoine Chehade et al. [13]	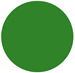	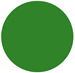	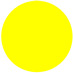	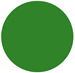	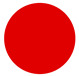	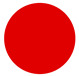	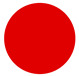	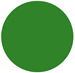
Antoine Chehade et al. [13]	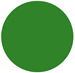	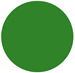	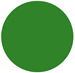	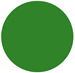	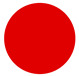	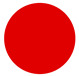	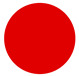	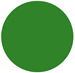
Antoine Chehade et al. [13]	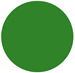	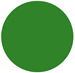	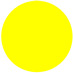	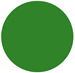	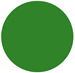	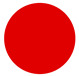	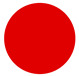	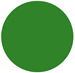
Antoine Chehade et al. [13]	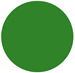	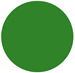	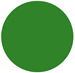	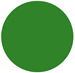	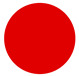	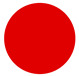	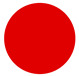	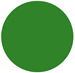
Antoine Chehade et al. [13]	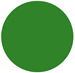	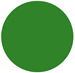	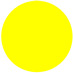	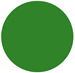	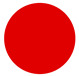	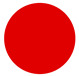	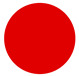	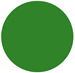
Fardal and Johannessen [14]	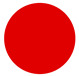	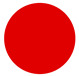	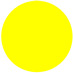	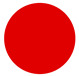	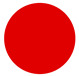	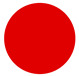	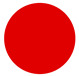	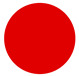
Yih, W. et al. [7]	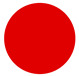	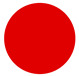	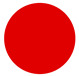	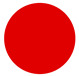	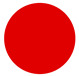	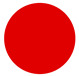	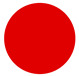	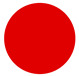
Yih, W. et al. [7]	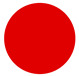	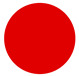	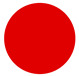	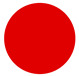	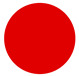	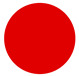	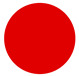	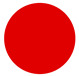
Yih, W. et al. [7]	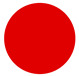	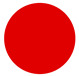	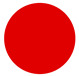	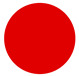	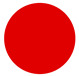	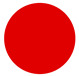	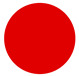	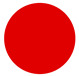
F. Ide et al. [15]	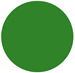	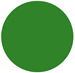	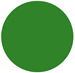	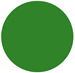	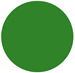	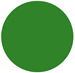	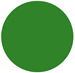	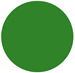
F. Ide et al. [15]	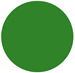	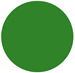	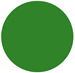	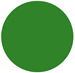	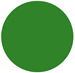	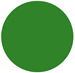	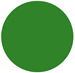	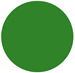
A.C. Chi et al. [16]	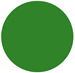	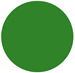	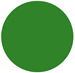	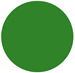	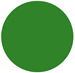	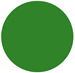	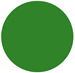	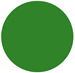
A.C. Chi et al. [16]	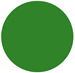	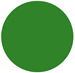	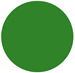	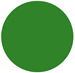	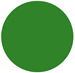	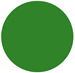	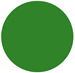	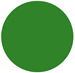
R.D. Preston et al. [17]	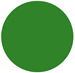	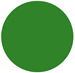	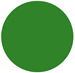	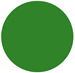	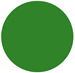	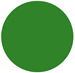	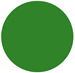	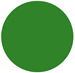
E. Mozaffari et al. [18]	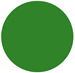	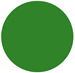	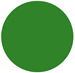	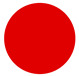	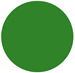	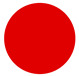	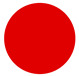	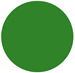
Usuii H. et al. [19]	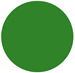	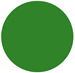	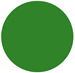	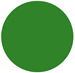	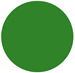	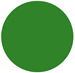	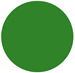	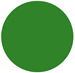
S E S Faustino et al. [20]	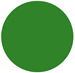	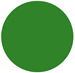	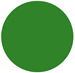	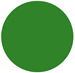	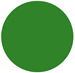	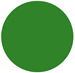	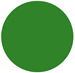	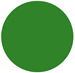
F. Ide et al. [21]	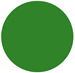	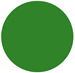	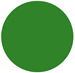	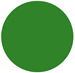	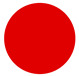	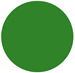	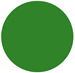	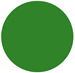
Jinbu, Y et al. [22]	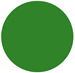	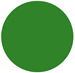	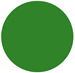	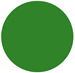	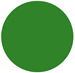	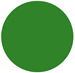	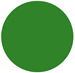	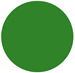
H. Vij et al. [23]	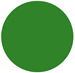	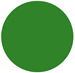	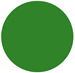	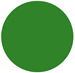	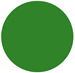	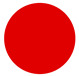	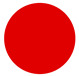	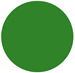
K. Sakamoto et al. [24]	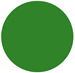	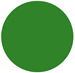	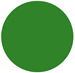	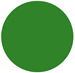	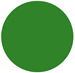	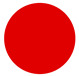	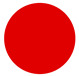	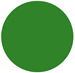
María del Carmen Vázquez-Romero et al. [2]	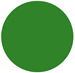	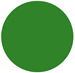	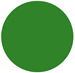	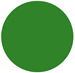	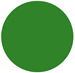	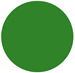	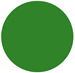	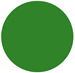
B.T. Rodrigues et al. [8]	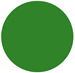	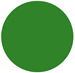	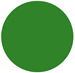	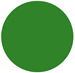	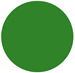	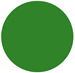	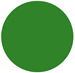	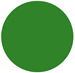
B.T. Rodrigues et al. [8]	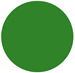	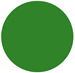	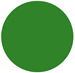	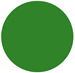	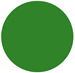	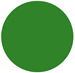	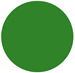	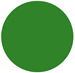
Lafuente-Ibáñez de Mendoza et al. [1]	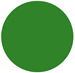	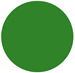	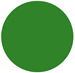	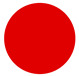	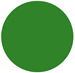	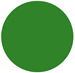	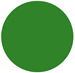	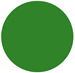
Lafuente-Ibáñez de Mendoza et al. [1]	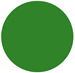	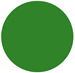	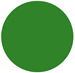	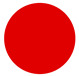	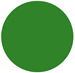	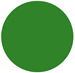	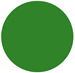	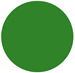
Lafuente-Ibáñez de Mendoza et al. [1]	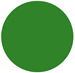	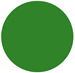	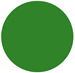	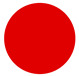	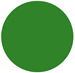	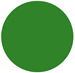	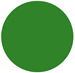	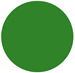
Lado Lako Loro et al. [25]	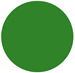	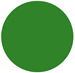	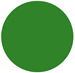	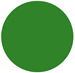	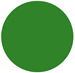	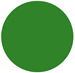	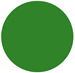	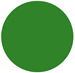
Lado Lako Loro et al. [25]	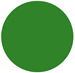	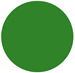	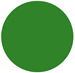	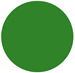	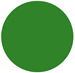	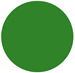	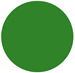	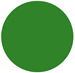
Brooks JK et al. [3]	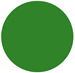	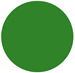	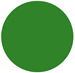	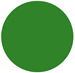	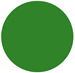	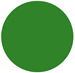	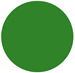	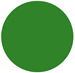
Kinaia et al. [19]	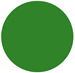	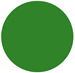	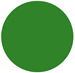	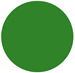	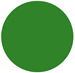	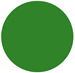	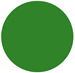	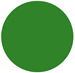
Kinaia et al. [19]	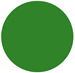	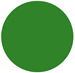	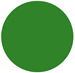	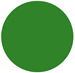	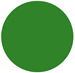	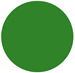	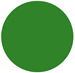	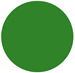

Q1: Were the patient’s demographic characteristics clearly described? Q2: Was the patient’s history clearly described and presented as a timeline? Q3: Was the current clinical condition of the patient on presentation clearly described? Q4: Were diagnostic tests or assessment methods and the results clearly described? Q5: Were the intervention(s) or treatment procedure(s) clearly described? Q6: Was the post-intervention clinical condition clearly described? Q7: Were adverse events (harms) or unanticipated events identified and described? Q8: Does the case report provide a takeaway lesson? 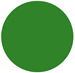 Yes, 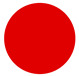 No; 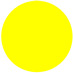 Unsure.

**Table 5:** Summary of the main clinicopathological features of lesions included in the differential diagnosis of POKC [32,33,40].

	POKC	LPA	Peripheral Giant Cells Granuloma	GCA	Inflammatory Cyst	Odontogenic Adenomatoid Tumor	Peripheral Ameloblastoma
**Clinical features**	Asymptomatic, slow-growing swelling of soft tissue	Small, asymptomatic, lateral to the roots of teeth	Firm, soft, nodular, or sessile or pediculate mass; surface occasionally ulcerated, bluish-red	Slow and painless swelling, usually solitary and small (measuring about 3–4 mm) nodules or vesicles, bluish	Painless or painful swelling in correspondence with a necrotic tooth (negative at vitality test)	Slow-growing, asymptomatic gingival swelling	Exophytic, sessile, solid gingival lesion (>1 cm); larger, more aggressive, with pain and swelling
**Radiographic appearance**	No evidence; sometimes uni or multi-loculated radiolucency	Unilocular radiolucency between the roots of vital erupted teeth, mostly premolars, canines and incisives; sometimes multilocular	Nonspecific, with foci of bone metaplasia in some cases; often widening of the periodontal ligament space	No bone involvement; possible bone resorption due to cyst pressure	Radiotransparent lesion encompassing one or more roots of a necrotic tooth	Resorption of the cortical cortex; tooth displacement without root resorption; in 78% of cases, radio-opacities	Bone expansion with corticated radiolucency
**Incidence**	12.5–13.6%	0.4–0.7 (of all cysts), 0.6–2.4% of odontogenic cysts	NR	3.2%	63.7% of all odontogenic cysts	3–7% of all odontogenic tumors	1–10% of all ameloblastoma
**Histology**	Uniform thin stratified squamous epithelium with a palisaded basal cell layer, superficial keratosis, and flat epithelial-connective tissue interface	Thin, nonkeratinized epithelium resembling reduced enamel epithelium. Epithelial lining exhibits focal thickenings or plaques, with clear glycogen-containing epithelial cells.	Non-encapsulated mass of tissue composed of a reticular and fibrillar connective tissue stroma containing abundant young connective tissue cells of ovoid or fusiform shape, and multinucleated giant cells	Subepithelial connective tissue wall covered by a thin, squamous or cuboidal epithelium with, in some points, glycogen-rich clear cells	Thin cell layers of nonkeratinized stratified squamous epithelium are associated with inflamed fibrous connective tissue and inflammatory infiltrates	Clusters of epithelial, fusiform or cuboidal cells, also with characteristics of duct-like spaces and epithelial bands with cancellous or cribriform configuration	Odontogenic epithelium organized in islands and chords, showing a follicular pattern and similar to the odontogenic islands of the central variant
**Localization**	Posterior mandibular region	Between the tooth roots, usually premolars	Interdental papilla, edentulous alveolar margin, or at the marginal gum level	Vestibular attached gingiva in the mandibular canine and first premolar areas	Necrotic teeth	Maxillary region, associated with osteo-included teeth (mostly canines)	Gingiva or alveolar crest

NR: not reported.

**Table 6:** Differences between intraosseous and peripheral subvariants [1,4].

	Intraosseous OKC	Peripheral OKC
*Gender*	Males	Females
*Age*	Wide range	Wide range
*Localization*	Posterior mandible	Anterior, upper maxilla
*Multiple presentation*	10%	0%
*Gorlin-Goltz Syndrome*	5–10%	9%
*Recurrence*	Up to 62%	12.5%
*Clinical aspect*	Small asymptomatic lesion	Asymptomatic, slow-growing swelling of soft tissue
*Radiographic appearance*	Well-defined unilocular radiolucency; in 20% of cases, multilocular radiolucency; 30% associated with an unerupted tooth	No evidence; either uni or multi-loculated radiolucency

## Data Availability

The data collected in the current study were downloaded from the following databases: PubMed (https://pubmed.ncbi.nlm.nih.gov; URL accessed 1 November 2024), Scopus (https://www.scopus.com; URL accessed on 1 November 2024), Web of Science (https://clarivate.com/academia-government/scientific-and-academic-research/research-discovery-and-referencing/web-of-science/; URL accessed on 1 November 2024), and Wiley Library (https://onlinelibrary.wiley.com; URL accessed on 1 November 2024).
